# Crosstalk of lncRNA and Cellular Metabolism and Their Regulatory Mechanism in Cancer

**DOI:** 10.3390/ijms21082947

**Published:** 2020-04-22

**Authors:** Yang-Hsiang Lin

**Affiliations:** 1 Liver Research Center, Chang Gung Memorial Hospital, Linkou, Taoyuan 333, Taiwan; yhlin0621@cgmh.org.tw; Tel.: +886-3-3281200 (ext. 7785); 2 International Program of Health Informatics and Management, Chang Gung University, Taoyuan 333, Taiwan

**Keywords:** lncRNA, glycolysis, mitochondria, cancer, therapeutic target

## Abstract

The imbalanced regulation of metabolic homeostasis and energy production is highly associated with inflammation, tumor growth, metastasis and cancer progression. Both glycolysis and oxidative phosphorylation maintain metabolic homeostasis and energy production in cells. Long noncoding RNAs (lncRNAs) are a class of non-protein-coding transcripts longer than 200 nucleotides. Furthermore, lncRNAs can function as either tumor suppressors or oncogenes in cancer. Dysregulated lncRNAs reportedly regulate cancer hallmarks such as tumor growth, metabolism and metastasis. Accordingly, uncovering the interaction between lncRNAs and cellular metabolism has become a necessity when attempting to identify effective therapeutic and preventive strategies in cancer progression. This review summarizes important knowledge of the actions of known lncRNAs-mediated cancer metabolism.

## 1. Introduction

Cancer cells can reprogram cellular metabolism to adapt and survive in nutritionally restricted environments [1]. Most cancer cells probably exhibit comprehensive reprogramming of glucose metabolism. Cancer cells prefer glycolysis over oxidative phosphorylation to generate adenosine triphosphate (ATP), regardless of oxygen abundance. Several studies have reported that tumor suppressors and oncogenes, such as p53, hypoxia-inducible factor-1α (HIF-1α) and Myc, can reprogram cellular metabolism in cancer cells [2,3,4]. Hexokinase 2 (*HK2*) is a well-known key gene for regulating glycolysis [5]. Multiple studies have indicated that HK2 is highly expressed in tumors and acts as a poor prognostic factor [6]. DeWaal and colleagues demonstrated that the inhibition of HK2 repressed glycolysis and induced oxidative phosphorylation in hepatomas [7]. Furthermore, the deletion of HK2 synergized with sorafenib to reduce tumor growth. These findings suggest that glycolysis is positively associated with cancer progression.

**Figure 1:**
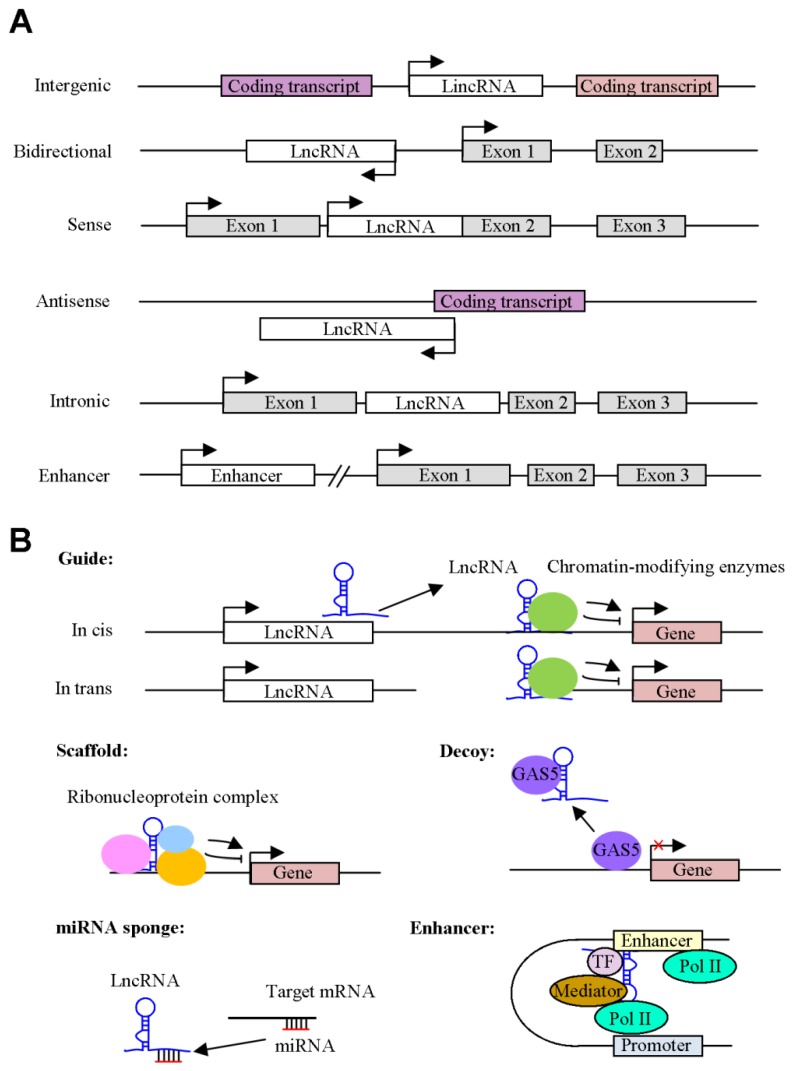
Classifications and actions of Long noncoding RNAs (lncRNAs) in cancer. (**A**) A schematic diagram indicating the classification of lncRNAs according to their orientation and position, including intergenic, bidirectional sense, antisense, intronic lncRNAs and enhancer RNAs (eRNAs). The arrow represents the transcription direction. (**B**) The mechanisms of lncRNAs. Guides are lncRNAs that can recruit specific proteins to target genes, either in cis or in trans. Scaffolds are lncRNAs that can associate with multiple proteins to form ribonucleoprotein complexes. This complex may modulate histone modifications such as methylation. Decoys are lncRNAs that can bind to transcription factors or other proteins and are subsequently removed from a specific location. LncRNAs can function as microRNA (miRNA ) sponges to regulate cellular function. Enhancers act as cis-acting elements and contribute to increase the target genes expression.

Long noncoding RNAs (lncRNAs) are a class of non-protein-coding transcripts longer than 200 nucleotides that are involved in multiple major biological functions, such as proliferation, apoptosis, metastasis, and cell metabolism [8]. LncRNAs can be transcribed by RNA polymerase II and undergo the splicing of multiple exons. LncRNAs can be categorized into six types depending on the origin of their expression (Figure 1A): (1) lncRNAs that can be transcribed between two coding transcripts (intergenic lncRNAs); (2) lncRNAs that can be transcribed from the promoter of a protein-coding gene but in the opposite direction (bidirectional lncRNAs); (3) lncRNAs that are expressed from the sense or antisense RNA strand of a protein coding gene (sense lncRNA or antisense lncRNA). These transcripts can entirely or partially overlap the exons of coding transcripts. Notably, natural antisense transcripts (NATs), a subset of lncRNAs, are transcribed from coding transcripts, including protein-coding genes and non-coding genes. NATs can be complementary to and overlap with coding transcripts; (4) lncRNAs that are expressed from the intron of a protein coding gene and do not overlap with any exon (intronic lncRNA); and (5) Enhancer RNA (eRNA) is an another class of nonconding RNA, which is transcribed from the enhancer. Moreover, lncRNA can fold and adopt the complex structures, resulting in interacting with DNA, RNA and proteins. A previous study reported that the 5′-end and 3′-end of HOX transcript antisense RNA (HOTAIR) can associate with polycomb repressive complex 2 (PRC2) and LSD1/CoREST/REST (RE1-silencing transcription factor) complex, respectively [9]. The structures of Metastasis Associated Lung Adenocarcinoma Transcript 1 (MALAT1), including hairpin regions, were specifically able to bind hnRNPG and hnRNPC proteins, which modulated gene expressions [10]. Collectively, the complex structures of lncRNA have flexibility, plasticity, and enable themself to regulate cellular functions. Functionally, five general mechanisms have emerged for broadly classifying lncRNA function (Figure 1B): (1) Guides are lncRNA transcripts that regulate target gene expression by binding to regulatory proteins, such as transcription factors and chromatin modifiers, to direct them to precise locations in the genome by *cis* or *trans* regulation. HOTAIR can guide the chromatin-modifying enzymes, such as PRC2, to bind to the target genes and regulates their expressions [11]. (2) Scaffolds are lncRNAs that act as the organizer and facilitate association with specific regulatory cofactors. The lncRNA, associated with the ribonucleoprotein (RNP) complex, is often important for RNP’s function. The scaffold lncRNAs, including MALAT1, TUG1 and ANRIL, interact with PRC1 or PRC2 and modulate target gene expression [12]. (3) Decoys are a class of lncRNAs that regulates specific gene expression by sequestering transcriptional regulators away from their binding site. Notably, growth arrest-specific 5 (GAS5) functions as decoy of nuclear protein, including glucocorticoid receptor and p53, and subsequently alter downstream target gene expression [13,14]. (4) The effect of lncRNA on miRNA sponges is that lncRNA regulates cellular function via complementary interacting with miRNA. The overexpression of lncRNA SNHG7 promoted cell proliferation, migration and invasion via sponging miR-216b [15]. (5) The eRNAs serve as trans-activating RNA and are transcribed from enhancer sequences, which cooperate with the promoter to modulate gene transcription. Zhang et al. [16] demonstrated that an eRNA, NET1-associated eRNA (NET1e), was upregulated in breast cancer. The overexpression of NET1e promoted cell proliferation. These observations indicated that NET1e acted as an oncogenic eRNA in breast cancer.

Crosstalk between lncRNAs and cellular metabolism has been implicated in cancer progression, and it is important to explore the nature of this connection. Interestingly, the energy stress-related lncRNA NBR2 (neighbor of BRCA1 gene 2) is induced by the liver kinase B1 (LKB1)/AMP-activated protein kinase (AMPK) axis [17]. The knockdown of lncRNA NBR2 inhibits AMPKα activity, resulting in unchecked cell cycle progression, altered autophagy/apoptosis response and enhanced tumor formation. Our group demonstrated that taurine upregulated gene 1 (TUG1) is highly expressed in hepatocellular carcinoma (HCC) specimens compared to adjacent normal tissues [18]. The knockdown of TUG1 promoted the marked inhibition of cell metastasis and glycolysis through the modulation of microRNA (miR)-455-3p. These findings suggest that lncRNAs are responsible for regulating cellular metabolism. In this review, we focus on how lncRNAs affect cellular metabolism through the modulation of specific pathways in cancers.

## 2. The Effects of lncRNAs on Glucose Metabolism in Cancer Cells

Glucose metabolism, including glucose uptake, oxidative phosphorylation and lactate production, is one of the energy sources of tumor cells [19]. Cancer cells reprogram glucose metabolism to enhance cell growth, survival, and drug resistance [20]. Under normal conditions, mitochondrial oxidative phosphorylation is the main source of ATP and produces 30 to 32 molecules of ATP. Notably, tumor cells tend to have increased glycolysis, which only produces two molecules of ATP, even under sufficient oxygen conditions. An important feature of this phenotype, the Warburg effect, is increased glucose uptake and fermentation of glucose to lactate [21]. ATP is formed faster than oxidative phosphorylation. Here, we summarize how lncRNAs modulate glucose metabolism via the regulation of glucose transporters (GLUTs) and glycolytic genes and how the lncRNA/GLUT or lncRNA/glycolytic gene axis controls tumorigenesis.

### 2.1. Glucose Transporter and lncRNA

**Figure 2:**
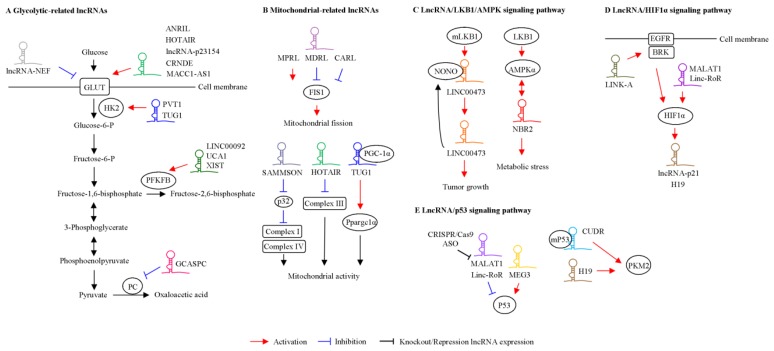
Functional roles of lncRNA in tumor metabolism. LncRNAs regulate target gene-mediated glucose metabolism (**A**) and mitochondrial function (**B**) in cancer. The role of lncRNA-mediated liver kinase B1 (LKB1)/AMP-activated protein kinase (AMPK) pathways (**C**), hypoxia-inducible factor 1α (HIF1α) (**D**), and p53 (**E**) in tumor cells was shown.

GLUTs play an important role in regulating glucose metabolism in tumor cells [22]. Several lncRNAs regulate the glycolytic pathway through the interaction of GLUTs (Figure 2A and Table 1). A novel large antisense noncoding RNA (ANRIL) is highly expressed in nasopharyngeal carcinoma (NPC) [23]. ANRIL promotes GLUT1 and lactate dehydrogenase A (LDHA) expression, resulting in the upregulation of glucose uptake and the promotion of cancer progression via the AKT/mTOR pathway. In HCC, HOTAIR promotes glycolysis by inducing GLUT1 expression and activating the mTOR signaling pathway [24]. Wang et al. demonstrated that lncRNA-p23154 promotes cell metastasis and glycolysis through the modulation of GLUT1 in oral squamous cell carcinoma [25]. In addition, the depletion of lncRNA NBR2 attenuates phenformin-induced glucose uptake and GLUT1 expression [17]. LncRNA-NEF is downregulated in patients with non-small-cell lung cancer [26]. The ectopic expression of lncRNA-NEF suppresses cell growth and glucose uptake via the inhibition of GLUT1 expression. Colorectal neoplasia differentially expressed (CRNDE) is upregulated in colorectal cancer and enhances GLUT4 expression and glucose uptake [27]. These findings indicate that the expression of GLUTs is regulated by lncRNAs and subsequently results in alterations in glycolysis. In addition to modulating the expression of GLUT1, lncRNAs also regulate the distribution of GLUT1 in tumor cells. LncRNA MACC1-AS1 is highly expressed under metabolic stress in gastric cancer cells [28,29]. MACC1-AS1 promotes the expression of GLUT1 surrounding the cell membrane. This finding suggests that MACC1-AS1 promotes glucose uptake and glycolysis by increasing the distribution of GLUT1 in the vicinity of the cell membrane. Collectively, lncRNA regulates glucose metabolism through the modulation of GLUTs expression in tumor cells. Moreover, understanding lncRNA-mediated regulation of GLUT protein subcellular localization is important not only in uncovering the function of individual proteins but also in developing therapeutic agents.

### 2.2. Glycolytic Enzyme and lncRNA

In addition to modulating GLUT expression or localization, several glycolytic genes are regulated by lncRNAs (Figure 2A and Table 1). HK can catalyze the first and irreversible step of glycolysis [30]. Five HK isoforms encoded by separate genes in mammalian cells have been identified [31]. HK1 is ubiquitously expressed in adult tissues, while HK2 is a more regulated form expressed in few adult tissues, including skeletal and cardiac muscle and adipose tissues [32], but it is highly expressed in cancer cells. In fact, HK2 acts as a key regulator of the Warburg effect in tumor cells. Lin et al. demonstrated that HK2 expression is regulated by the TUG1/miR-455-3p/AMPKβ2 axis and is involved in TUG1-mediated function in HCC [18]. As indicated by a previous study, TUG1 is associated with HK2-mediated glycolysis in osteosarcoma [33]. LncRNA PVT1 plays an oncogenic role in multiple cancers [34]. The overexpression of PVT1 enhances glucose metabolism via modulation of the miR-497/HK2 axis in osteosarcoma [35]. Pyruvate kinase M2 (PKM2) is a metabolic enzyme that plays a crucial role in cellular metabolism and tumor growth [36]. Li et al. demonstrated that miR-675 promotes lncRNA H19 expression through the activation of the early growth response protein 1 (EGR) pathway in hepatoma cells [37]. Moreover, H19 induces PKM2 expression, which is involved in the Warburg effect during cancer progression. Pyruvate carboxylase (PC) is involved in modulating cellular metabolism, including glucose metabolism, gluconeogenesis and de novo fatty acid synthesis [38]. LncRNA gallbladder cancer–associated suppressor of pyruvate carboxylase (GCASPC) is downregulated in gallbladder cancer specimens and correlated with cancer progression [39]. Furthermore, GCASPC can interact with PC and then inhibit its protein expression via the modulation of protein stability. The expression levels of 6-phosphofructo-2-kinase/fructose-2,6-biphosphatase 2 (PFKFB2), which is involved in glucose metabolism, are regulated by lncRNAs, including LINC00092, UCA1, and X inactive-specific transcript (XIST) [40,41,42]. For example, a high level of CXCL14 in cancer-associated fibroblasts (CAFs) mediates the upregulation of LINC00092 in ovarian cancer cells [40]. Mechanistically, LINC00092 is associated with PFKFB2, thereby inducing cell metastasis by modulating glycolysis in cancer-associated fibroblasts (CAFs). These findings suggest that crosstalk between tumor cells and stromal cells mediated by LINC00092 is important in cell metastasis and glycolysis. Recently, lncRNA XIST was found to act as a competing endogenous RNA (ceRNA) that regulates PFKFB2 expression by sponging miR-212-3p and miR-122-5p [42]. Accordingly, lncRNA-mediated glucose metabolism may occur through three different mechanisms: (1) alteration of the expression levels of GLUT/glycolytic enzymes, (2) alteration of the distribution of GLUTs, or (3) interactions with glycolytic genes and the modulation of their activity. These observations suggest that lncRNAs act as upstream regulators of glucose metabolism and that they may be used for novel therapeutic target development to prevent cancer progression.

## 3. The Effects of lncRNAs on Mitochondrial Function in Cancer Cells

Mitochondria contain a membrane bilayer that consists of an inner and an outer mitochondrial membrane. Mitochondria are responsible for producing the majority of cellular energy (ATP) through the process of oxidative phosphorylation (OXPHOS), Krebs cycle and the synthesis of biosynthetic precursors, such as nucleotides, amino acids, lipids, heme and nicotinamide adenine dinucleotide phosphate hydrogen (NADPH) [43,44]. Accordingly, mitochondrial quality control is pivotal for cancer maintenance and progression. Mitochondria are highly dynamic organelles accompanied by fusion and fission, which modulate mitochondrial shape and function. Thirteen structural subunits of complexes I, III, IV, and V, 2 rRNAs, and 22 tRNAs are encoded by the mitochondrial genome [45]. The other components of OXPHOS are encoded by the nuclear genome. Increasing evidence indicates that lncRNAs encoded by nuclear DNA and mitochondrial DNA are correlated with mitochondrial functions. These associations are described below and shown in Figure 2B and Table 1.

### 3.1. Nuclear DNA-Encoded lncRNAs and Mitochondrial Function

MiRNA processing-related lncRNA (MPRL) is highly expressed in tongue squamous cell carcinoma cell lines after cisplatin treatment [46]. MPRL alters mitochondrial fission and cisplatin sensitivity via the interaction of pre-miR-483. This association represses the expression of the mature form of miR-483-5p and then upregulates the miR-483-5p target gene mitochondrial fission 1 (FIS1), which is responsible for regulating mitochondrial fission. Mitochondrial dynamic related lncRNA (MDRL) reduces miR-361 expression and indirectly induces the expression of miR-484, a negative regulator of FIS1 protein expression [47]. Mechanistically, miR-361 associates with pri-miR-484 and suppresses its maturation via Drosha in the nucleus, resulting in increased FIS1 expression and cell apoptosis. Thus, mitochondrial fission and apoptosis are indirectly regulated by MDRL. Another study indicated that lncRNA, named cardiac apoptosis-related lncRNA (CARL), repressed mitochondrial fission and apoptosis by targeting miR-539 and PHB2 [48]. Thus, lncRNA can impact mitochondria quality control involved in cancer progression through the modulation of miRNA expressions.

Peroxisome proliferator-activated receptor gamma coactivator 1α (PGC-1α) is an important transcriptional coactivator that modulates mitochondrial biogenesis and mtDNA replication [49,50]. Ppargc1α gene is upregulated by TUG1 in podocytes [51]. Mechanistically, TUG1 directly interacts with PGC-1α protein and contributes to induce the binding of PGC-1α to its own promoter. Furthermore, the TUG1 binding region upstream of the Ppargc1α gene was identified by genome-wide chromatin isolation by RNA purification sequencing. This finding indicates the important regulatory mechanism of lncRNA and mitochondria in a murine model. Additionally, several lncRNAs have been reported to control OXPHOS activity through the modulation of the OXPHOS complex subunit. HOTAIR, involved in OXPHOS activity, is highly expressed and an unfavorable prognosis in a variety of cancers [52]. The knockdown of HOTAIR leads to altered mitochondrial morphology in HeLa cells, characterized by mitochondrial swelling and loss of cristae. Additionally, HOTAIR can reduce OXPHOS complex III subunit VII protein expression, which functions as a regulator of OXPHOS [53]. Kong and colleagues demonstrated that the depletion of HOTAIR promoted the apoptosis of head and neck squamous cell carcinoma through the induction of mitochondrial calcium uptake 1 (MICU1) [54]. Mitochondrial membrane potential was altered by HOTAIR blockage. UCA1 enhanced mitochondrial function through the upregulation of ARL2, which plays an important role in mitochondrial activity [55]. Survival associated mitochondrial melanoma specific oncogenic non-coding RNA (SAMMSON) is an intergenic lncRNA that is particularly expressed in melanoma cells [56]. SAMMSON is expressed in the cytoplasm and mitochondria. SAMMSON can bind to complement C1q-binding protein p32, which is required for mitochondrial pre-rRNA processing [57]. Functionally, SAMMSON silencing reduced p32 expression, leading to a decrease in OXPHOS complex I and IV activity and mitochondrial membrane depolarization. The knockdown of p32 leads to a fragmented mitochondrial network and shifts tumor cell metabolism from OXPHOS to glycolysis [58]. In line with activity assays, complex I and complex IV subunit protein levels were reported to be reduced, while complex II subunit levels were unchanged. The A lncRNA RNA component of the RNA processing endoribonuclease (RMRP) can be transcribed by nuclear DNA and associated with RNA binding proteins (HuR and GRSF1) and translocate it to mitochondria [59]. The depletion of GRSF1 reduces the mitochondrial levels of RMRP, resulting in decreased oxygen consumption rates and mitochondrial DNA replication. These investigations provide a new insight into how RNA binding proteins influence the localization of lncRNAs, where they exert their normal function.

### 3.2. Mitochondrial DNA-Encoded lncRNAs and Mitochondrial Function

Mitochondria are able to take up lncRNAs encoded by nuclear DNA but can also encode lncRNAs with their own DNA. Three lncRNAs, *lncND5*, *lncND6* and *lncCytB*, transcribed from mtDNA, have been reported by deep-sequencing results [60]. A previous study reported that mitochondrial RNase P protein 1 (MRPP1) and pentatricopeptide repeat domain protein 2 (PTCD2) are involved in the regulation of the expression levels of the mature form of tRNA, rRNA and mitochondrial gene expression [60]. Notably, MRPP1 and PTCD2 can regulate lncND5, lncND6 and lncCytB expression. These observations suggest that important contributions of these nuclear-encoded proteins impact mitochondrial-related lncRNAs. However, the detailed regulatory mechanism should be fully addressed. Chimeric lncRNAs containing mtDNA-encoded genes have been characterized. Sense mitochondrial ncRNA (SncmtRNA) is expressed in proliferating cells, including normal and cancer cells, but not in resting cells [61,62]. SncmtRNA has a characteristic RNAse-resistant double-stranded structure with a 40-nucleotide loop. This association between cell growth and the expression of SncmtRNA-1 suggests a functional role for this transcript in cell cycle progression. Recently, anti-sense SncmtRNA (ASncmtRNA) is downregulated by HPV-16 or 18 in human immortalized keratinocytes. Moreover, the E2 oncogene is involved in this regulation [61]. Fitzpatrick et al. demonstrated that the knockdown of ASncmtRNA induced cell death and inhibited tumor growth via cell cycle-related genes, such as cyclin B1, cyclin D1, CDK1, CDK4, and surviving in breast cancer cell lines [62]. Thus, these findings shed light on the functional role of ASncmtRNA in cancer progression.

## 4. The Effects of lncRNAs on Oxidative Stress in Cancer Cells

Oxidative stress (OS) is indicated by the homeostatic imbalance of antioxidant and free radicals in cells [63]. In particular, free radicals such as reactive oxygen species (ROS) and reactive nitrogen species (RNS) act as contributor to control OS. Indisputably, OS contributes to cell death and physiological dysfunction, which could be ascribed to DNA damage and inflammation. Recent studies have revealed that lncRNAs function as mediators for negatively or positively regulating OS in cancer cells (Table 1). The nuclear factor erythroid 2-related factor 2/Kelch-like ECH-associated protein 1/antioxidant response element (Nrf2/Keap1/ARE) axis is an important executer in response to OS to maintain the balance of the oxidation/antioxidant system [64]. Chen’s group indicated that NEAT1 was highly expressed in sepsis-induced acute kidney injury (AKI) patients. The knockdown of NEAT1 alleviated lipopolysaccharide-induced injury in rat mesangial cells [65]. Additionally, NEAT1 repressed OS-induced vascular endothelial cell injury by activating the miR-181d-5p/CDKN3 pathway [66]. Several lncRNAs have been shown to be associated with cisplatin resistance. Zhang et al. demonstrated that H19 was upregulated in cisplatin-resistant cells compared with parental cells [67]. The knockdown of H19 in cisplatin-resistant cells resulted in the recovery of cisplatin sensitivity in vitro and in vivo. Moreover, NRF2-regulated genes, including NQO1, GSR, G6PD, GCLC, GCLM and GSTP1, which are involved in the glutathione metabolism pathway, were repressed in H19-depleted cells. Thus, the crosstalk of H19 and glutathione metabolism may regulate cancer-drug resistance. In addition, the ectopic expression of H19 suppressed inflammation and OS in a rat model of diabetic cardiomyopathy (DCM). Moreover, H19 expression was suppressed in high-glucose conditions. The depletion of H19 resulted in decreased miR-657 expression. The expression of voltage-dependent anion channel 1 (VDAC1) was upregulated in the inhibition of miR-657 cell lines, which contributed to enhance cell apoptosis [68]. Notably, the overexpression of H19 suppressed VDAC1 expression and repressed apoptosis under a high glucose condition. These findings indicated that the H19/miR-657/VDAC1 axis is responsible for modulating high glucose-induced apoptosis, suggesting that it may establish a novel therapeutic target for DCM. Metastasis-associated lung adenocarcinoma transcript 1 (MALAT1) plays a negative or positive role under OS. The overexpression of MALAT1 plays a positive role in the antioxidant pathway by inducing hydrogen peroxide (H_2_O_2_) in human umbilical vein endothelial cells (HUVECs) [69]. Mechanistically, Keap1 expression levels are repressed by MALAT1, leading to the activation and stabilization of the Nrf2 protein, resulting in the attenuation of OS-mediated damage and lipid peroxidation in H_2_O_2_-treated HUVECs. MALAT1 has also been identified as an Nrf2 regulator that associates with Nrf2 [70]. In addition, MALAT1 modulates apoptosis and oxidative stress via the p38MAPK pathway in human lens epithelial cells [71]. These findings suggest that actions on the Nrf2/Keap1/ARE pathway might be an important strategy for the lncRNA-mediated regulation of OS.

## 5. The Effects of lncRNAs/Signal Transduction Pathways on Cellular Metabolism in Cancer Cells

Numerous studies indicate that cellular metabolism is regulated by lncRNAs and their downstream signaling pathways, including the LKB1/AMPK, HIFα and p53 pathways. This crosstalk is described below and shown in Figure 2C–E.

### 5.1. LKB1/AMPK Signaling Pathway

AMPK is an energy sensor and is required for glucose homeostasis [72]. Upon the activation of AMPK, it enhances the activation of the TSC2 complex, leading to the inhibition of the mTOR-activated GTP-binding protein Rheb. Under metabolic stress, the AMPK-mediated phosphorylation of TSC2 protects cells from apoptosis. Liver kinase B1 (LKB1), known as a protein kinase, acts as a tumor suppressor that regulates cell proliferation and energy metabolism by regulating the activity of mTOR [73]. LKB1 is an upstream regulator of AMPKα, and therapy induces the phosphorylation of AMPK. The knockdown of LKB1 enhances tumor growth, glucose uptake, ATP production, and macromolecules synthesis. Notably, in LKB1-deficient cancer cell lines, the metabolic reprogramming effect is regulated by HIF-1α, which executes its antagonism by suppressing mTORC1 [74]. LINC00473 is upregulated in human non-small cell lung cancer and is significantly correlated with LKB1 activity [75]. Mechanistically, nuclear LINC00473 associates with non-POU domain-containing octamer-binding protein (NONO), which is involved in the cAMP signaling pathway. Glucose starvation promotes the activation of AMPK or acetyl-CoA carboxylase [76]. LncRNA NBR2 is induced by the LKB1/AMPK signaling pathway under metabolic stress (glucose starvation) [17]. NBR2 is a tumor suppressor that enhances the activity of AMPKα. The depletion of NBR2 significantly attenuates the phosphorylation of AMPK and mTORC1 inactivation, suggesting the regulation of the NBR2/AMPKα feedback loop mechanism. Notably, these lncRNAs were involved in facilitating metabolic plasticity by regulating the LKB1/AMPKα axis. 

### 5.2. Hypoxia

HIF acts as a transcription factor that is expressed by tumor cells adapting to hypoxic environments [77]. The activation of HIF-1α induces the Warburg effect, partly through the upregulation of GLUTs and glycolytic enzymes or the suppression of OXPHOS [78,79]. Under hypoxic conditions, lincRNA-p21 is upregulated by HIF-1α and then enhances HIF-1α protein stability, establishing a positive feedback regulation [80]. Functionally, lincRNA-p21 is involved in hypoxia-mediated glycolysis. In normal liver cell line L02, the glycolytic genes (GLUT4, HK2 and ENO-1), MALAT1 and HIF-α are upregulated by arsenite treatment [81]. Moreover, MALAT1 enhances arsenite-induced glycolysis by inducing the disassociation of HIF-1α from VHL, preventing the VHL-mediated ubiquitination of HIF-1α. In addition, hypoxia induces lncRNA H19 expression, which is involved in hypoxia-induced signal transduction processes in cancer cells, resulting in modulating glucose metabolism [82]. Lin et al. demonstrated that an lncRNA in the cytoplasm, long intergenic noncoding RNA for kinase activation (LINK-A), is responsible for regulating metabolic reprogramming in triple-negative breast cancer [83]. Mechanistically, LINK-A induces the recruitment of BRK to the EGFR-GPNMB complex and subsequently enhances the breast tumor kinase (BRK) activity. Moreover, the phosphorylation of HIF-1α at tyrosine 565 by BRK and then stabilizes HIF-1α via interfering with the hydroxylation of proline 564. Takahashi and colleagues reported that linc-ROR is associated with hypoxic conditions and acts as a molecular sponge of miR-145 to regulate HIF-1α expression [84]. The underlying crosstalk of lncRNA and HIF-1α in cancer progression may lead to clinical applications.

### 5.3. p53 Signaling Pathway

The loss of p53 in the cell can lead to mitochondrial respiratory damage and increased glycolysis [85]. Glucose transporters, such as GLUT1 and GLUT4, and glycolytic genes, including phosphoglycerate mutase (PGM), TP53-induced glycolysis and apoptosis regulator (TIGAR) and 6-phosphofructokinase 1 (PFK1), are regulated by p53. Several lncRNAs are directly or indirectly regulated by p53. Wu et al. demonstrated that mutant p53 (N340Q/L344R) facilitates the progression of HCC by upregulating PKM2 [86]. LncRNA CUDR is associated with the mutated form of p53. This complex binds to the promoter regions of PKM2 and enhances its gene expression. The overexpression of maternally expressed gene 3 (MEG3) induces p53 expression and the activation of p53 downstream target genes [87,88]. Notably, an antisense transcript of p53 (wrap53) modulates p53 expression through targeting the 5′ UTR of p53 [89]. Tripathi et al. reported that the knockout of MALAT1 in fibroblasts stimulates DNA damage repair and results in the activation of p53 and its downstream target genes [90]. Upon DNA damage, lincRNA ROR acts as a repressor of p53 [91]. Accordingly, the p53-mediated glucose metabolism in cancer progression is manifested in the suppression of glycolysis and the promotion of oxidative phosphorylation and tricarboxylic acid (TCA) cycle.

## 6. Application of lncRNA for the Treatment of Cancer

Tumor formation is a complicated process. The approaches in cancer treatment can be performed by chemotherapy, targeted therapy and immunotherapy. Therefore, the cancer treatment requires understanding target genes involved in cancer progression for successful intervention. As mentioned above, several lncRNAs expressions are dysregulared in cancers and involved in metabolic reprogramming and cancer progression. Because of the tissue-specific characteristics of lncRNAs, they could be the next generation of biomarkers for human cancer or disease. LncRNAs may be promising targets for treating cancer. Although the therapeutic approach of lncRNAs still remains at the laboratory stage, several types of approaches targeting lncRNAs in cancer treatment are described below.

### 6.1. CRISPR/Cas9 Genome Editing Technique

Recently, CRISPR/Cas9 has also received extensive attention in the treatment of cancer [92]. One of the limitations for knockout lncRNAs by CRISPR/Cas9 system is that nucleotide insertions/deletions mutation may not lead to a functional loss of lncRNAs, which lack an open reading frame. To solve this challenge, Ho et al. [93] demonstrated that, via the adopted unique selection system, a marker gene could be integrated into the genome via homologous recombination. In fact, lncRNA-21A, UCA1 and AK023948 were successfully knockout in cancer cell lines using CRISPR-Cas9. In addition, Daneshvar and co-workers demonstrated that a sequence encoding GFP followed by a poly (A) signal was inserted to the downstream of the Divergent to GSC, induced by TGF-β family signaling (DIGIT) transcription start site using CRISPR-Cas9 system [94]. The insertion of cassette allowed for the induction of transcription of GFP at the DIGIT locus but led to termination at the poly(A) signal, resulting in disrupting full length DIGIT expression. Several studies have shown that CRISPR/Cas9 can successfully inhibit lncRNA expression by targeting the transcriptional site of a gene promoter to repress transcription in tumor cells and animal models [95]. Another successful strategy for knockout lncRNA is that TATA box of NEAT1 was removed from the original region by CRISPR-Cas9-mediated homologous recombination [96]. An lncRNA named gastric cancer metastasis associated long noncoding RNA (GMAN) is highly expressed in gastric cancer and is associated with poor prognosis [97]. A well-designed animal experiment indicated that targeting *GMAN* via the CRISPR/Cas9 system significantly repressed the metastasis of gastric cancer cells and improved overall survival in a mouse model. Alternatively, CRISPR activation (CRISPRa) can selectively induce a gene of interest from its original chromosomal locus [98]. This methodology was applied to activate protein-coding and noncoding genes to explore their biological function in cells. This system requires catalytically dead Cas9 (dCas9) protein fused to the VP64 transcriptional activator and a single-guide RNA (sgRNA) targeted to the promoter region of the gene of interest. The activation of TUG1 by CRISPRa partially restores the thyroid hormone-inhibited cell growth effect in hepatoma cell lines [99]. Some lncRNAs are expressed specifically in different tissues and even different people. Thus, personalized treatment depending on the situation of patients can be performed in the future. So far, CRISPR/Cas9 has broad adaptability and target specificity. However, the off-target effects of CRISPR/Cas9 still exist in practical applications. Therefore, oncologists should be more cautious in designing gene-editing therapies. Currently, the clinical application of the CRISPR/Cas9 system in targeting lncRNAs to treat cancer is not well understood. Moreover, developing more specific gene-editing tools is important.

### 6.2. Antisense Oligonucleotides

Antisense oligonucleotides (ASOs) have been used in clinical applications to target mRNAs involved in cancer progression [100]. Basically, ASOs, which form a DNA–RNA structure with a target, trigger RNase-H-mediated RNA degradation. In addition, ASOs can bind to splicing regulatory sequences and modulate the splicing pathway. Notably, the exact sequences of lncRNAs expressed in tumor cells or tissues remain unclear. The variants of lncRNAs can be identified using the rapid amplification of cDNA ends (RACE) assay. Accordingly, targeting lncRNA by the ASO strategy may be a promising method for cancer therapy. In addition, ASOs have been shown to specifically alter RNA splicing events. These ASOs can bind to splicing regulatory sequences and modulate the splicing pathway. The therapeutic repression of NATs such as *ANRIL* and *p21-AS* by treating with a specific ASO can induce the overlapping tumor suppressor genes expression [101,102]. The knockdown of ASncmtRNA by ASO resulted in the dramatic inhibition of cell proliferation and tumor growth in vitro and in a xenograft model [62]. Gone et al. demonstrated that specific ASO-MALAT1 treatment reduced its expression in lung cancer cells and inhibited cell migration ability [103]. Similar effects were observed in breast cancer [104], providing ideas for the clinical treatment of cancer progression. Because of the poor membrane permeability of ASOs, ASOs are mainly confined to the cytoplasm. Thus, it is difficult for ASOs to modulate nuclear lncRNA expression.

### 6.3. Short Hairpin RNAs

Gene silencing via the RNA interference (RNAi) holds great potential for the treatment of various diseases, such as cancer [105]. The delivery methods can be executed by transfecting plasmid that express short hairpin RNAs (shRNAs). On the other hand, the delivery of shRNA-expressing plasmid by viral vector has the advantage of long-term treatment. There is much evidence on the use of shRNAs to target lncRNAs to prevent cancer progression. Our group demonstrated that the knockdown of TUG1 or BC200 with shRNAs in hepatoma reduced cell proliferation [18,106]. In addition, transfecting HOTAIR-specific shRNA via a retrovirus in a breast cancer cell line signifi-cantly inhibited tumor cell metastasis in vivo [107]. It was also found that the knockdown of lncRNA-PNUTS via an adenovirus system could reduce the epithelial–mesenchymal transition and metastases [108]. The knockdown of lncRNA-BCAR4 by lentiviral transfection significantly inhibited the formation of metastases in breast cancer in vivo in mice [109]. Adeno-associated viruses (AAVs) are structures of uncoated, single-stranded DNA [110]. The application of adenovirus vectors is far more extensive, and there are some clinical trials [111,112]. AAV-based vectors are an efficient gene delivery system, mainly because they are non-pathogenic, do not elicit an immune response and are stable in live cells [110]. After large-scale screening, ideal AAV systems have been developed to target human cancer cells [113]. AAVs have laid a solid foundation for the clinical treatment of tumors by targeting lncRNAs. So far, Patient-Derieved Xenograft Models (PDX) models are a useful model for pre-clinical investigation. Some studies reported that PDX has been applied to identify lncRNAs function from basic research to pre-clinical studies [114]. The therapeutic responses of SAMMSON in vivo were tested in PDX melanoma models (designated Mel006 and Mel010) [56]. The silence of SAMMSON in Mel006 and Mel010 xenografts significantly increased apoptosis and repressed the tumor growth. Notably, this assay in the PDX model did not cause weight loss or any relevant adverse effects. Thus, the depletion of SAMMSON is highly efficient and useful for anti-melanoma. Recently, the association of lncRNA-p21 and neuroendocrine prostate cancer cells (NEPC)-PDX has been uncovered [115]. The expression levels of lncRNA-p21 are highly expressed in NEPC. Moreover, enzalutamide (Enz) treatment enhanced the lncRNA-p21 to promote the neuroendocrine differentiation (NED). Furthermore, the treatment of the EZH2 inhibitor suppressed the Enz-induced NED in the PDX model. Together, these findings may provide some potential therapies of treating EZH2 inhibitors to target the lncRNA-p21/EZH2 signaling pathway. Several studies support that it is worth utilizing PDXs model to investigate targeted therapies for cancer progression with the integration of clinical observations.

## 7. Conclusions

Recently, reprogramming cellular metabolism has become a hallmark of cancer cells. Increasing studies have elucidated that lncRNAs, which were originally regarded as genomic junk, are pivotally involved in tumor growth, metastasis, metabolism and cancer progression. The lncRNA networks modulating cellular metabolism in cancer are comprehensively listed in Table 1. Theoretically, these effects are highly associated with metabolism, which can impact the regulation of cellular metabolism and energy homeostasis. In this review article, we highlight the lncRNAs associated with glucose metabolism, mitochondrial function and oxidative stress and present their potential mechanisms. However, some lncRNAs exert opposite roles in different studies, which might be due to the tissue-specific features of the lncRNAs. As discussed in this review, these findings suggest that these metabolic lncRNAs may provide a novel approach for the diagnosis and treatment of cancer progression. Further studies are required to address the detailed mechanisms of lncRNAs, and cellular metabolisms are required.

## Figures and Tables

**Table 1:** Metabolic-regulated lncRNAs and their potential mechanisms in cancers.

Gene Name	Principal Functions	Molecules and Signaling Pathways Involved^a^	Cancer Development	Prognostic Markers in Cancer^b^	Up- or Downregulation^c^	Cancer/Cell Types	Reference
*ANRIL*	Glucose uptake	GLUT1, LDHA, AKT/mTOR	Progression	✓	Up	NPC	[23]
*HOTAIR*	Glycolysis Mitochondrial function Apoptosis	GLUT1, mTOR, vimentin, MICU1	Progression	✓	Up	HCC, HeLa cell, Head and neck squamous cell carcinoma	[24,53,54]
*LncRNA-p23154*	Glycolysis Metastasis	GLUT1	Progression	✓	Up	Oral squamous cell carcinoma	[25]
*LncRNA NBR2*	Glucose uptake Tumor growth Apoptosis	GLUT1, AMPK activity, mTORC1	Regression	✓	Down	786-O, MDA-MB-231	[17]
*LncRNA-NEF*	Cell growth Glycolysis	GLUT1	Regression	✓	Down	Non-small-cell lung cancer	[26]
*CRNDE*	Glucose uptake Warburg effect	GLUT4, insulin/IGF axis	Progression	✓	Up	Colorectal cancer	[27]
*MACC1-AS1*	Glycolysis Cell viability Stemness	AMPK/Lin28, TGFβ1, miR-145-5p	Progression	✓	Up	Gastric cancer	[28,29]
*TUG1*	Tumor formation Glycolysis Metastasis OXPHOS	HK2, miR-455-3p, AMPKβ2, PGC-1α	Progression	✓	Up	HCC, Immortalized mouse podocytes	[18,51]
*PVT1*	Glycolysis Cell growth Cell cycle Invasion	miR-497, HK2	Progression	✓	Up	Osteosarcoma	[35]
*H19*	Warburg effect Drug resistance Glutathione metabolism	miR-675, PKM2, EGR pathway NRF2, miR-657	Dual role	✓	Dual role	Liver cancer, diabetic mouse model	[68,82]
*GCASPC*	Cell growth Tumor formation	miR-17-3p, PC	Regression	✓	Down	Gallbladder cancer	[39]
*LINC00092*	Glycolysis Migration	PFKFB2, CXCL14	Progression	✓	Up	Ovarian cancer	[40]
*LncRNA XIST*	-	PFKFB2, miR-212-3p, miR-122-5p, AMPK	-	-	-	Acute kidney injury	[42]
*MPRL*	Mitochondrial fission Apoptosis	E2F1, miR-483-5p, FIS1	Regression	✓	Up (in chemosensitive patient)	Tongue squamous cell carcinoma	[46]
*MDRL*	Mitochondrial fission Drug resistance Apoptosis	miR-484, miR-361	-	-	-	Mouse cardiomyocyte	[47]
*CARL*	Mitochondrial fission Apoptosis	miR-539, PHB2	-	-	-	Mouse cardiomyocyte	[48]
*UCA1*	Mitochondrial function Colony formation Tumor growth	miR-195/ARL2	Progression	✓	Up	Bladder cancer	[55]
*SAMMSON*	Mitochondrial homeostasis Colony formation	P32, MAPK, complex I/IV	Progression	✓	Up	Melanoma	[56]
*RMRP*	Oxygen consumption Mitochondrial DNA replication	HuR, GRSF1	-	-	-	Hela, HEK293 cells	[59]
*ASncmtRNA*	Tumor growth Cell death	Cyclin B1, cyclin D1, CDK1, CDK4, survivin	Progression	-	-	Breast cancer	[62]
*NEAT1*	Oxidative stress	miR-204, NFκB, miR-181d-5p/CDKN3 axis	Progression	✓	Up	Rat mesangial cells, endothelial cells	[66,70]
*MALAT1*	Oxidative stress Antioxidant Lipid peroxidation Apoptosis	KEAP1, NRF2, p38/MAPK	Progression	✓	Up	HUVEC, lens epithelial cells	[69,70]
*LncRNA-p21*	Hypoxia Glycolysis Warburg effect Tumor formation	HIF-1α, VHL	Progression	✓	Up	HeLa, MCF7, H1299, IMR90	[80]
*LINK-A*	Metabolic reprogramming	BRK, EGFR, GPNMB	Progression	✓	Up	Triple-negative breast cancer	[83]
*Linc-RoR*	Hypoxia	RPS6KB1, PDK1, HIF-1α, miR-145, p53	Progression	✓	Up	Liver cancer	[84,91]
*LINC00473*	Tumor growth	LKB1, CRTC1, CREB, NONO	Progression	✓	Up	Lung cancer	[75]
*lncRNA CUDR*	Tumor growth	PIM1, PKM2, p53	Progression		Up	Liver cancer	[86]

a: Downstream molecules and signaling pathways involved in lncRNA-mediated functions. b: ✓: Target gene acts as a prognostic marker in cancer. -: Information is unavailable. c: Up: LncRNA is upregulated in cancer compared with adjacent normal tissues. Down: LncRNA is downregulated in cancer compared with adjacent normal tissues.
